# Development and Evaluation of Minocycline Hydrochloride-Loaded In Situ Cubic Liquid Crystal for Intra-Periodontal Pocket Administration

**DOI:** 10.3390/molecules23092275

**Published:** 2018-09-06

**Authors:** Zhuanzhuan Yang, Xin Liang, Xiaojing Jiang, Jian Guo, Yaotian Tao, Shengmei Wang, Yingji Cao, Shuangying Gui

**Affiliations:** 1 Department of Pharmaceutics, College of Pharmacy, Anhui University of Chinese Medicine, Hefei 230012, China; zhuimengyzz@126.com (Z.Y.); xinl0626@hotmail.com (X.L.); 18355180342@163.com (X.J.); guoj0719@126.com (J.G.); telen1124@yeah.net (Y.T.); sinmay_w@163.com (S.W.); xiazi2006@126.com (Y.C.); 2 Institute of Pharmaceutics, Anhui Academy of Chinese Medicine, Hefei 230012, China

**Keywords:** minocycline hydrochloride, in situ cubic liquid crystal, chronic periodontitis, sustained-release formulation, local delivery system

## Abstract

In the present study, an injectable in situ liquid crystal formulation was developed for local delivery of minocycline hydrochloride (MH) for chronic periodontitis treatment. The physicochemical properties, phase structures, in vitro drug release and pharmacodynamics of in situ liquid crystals were investigated. The optimal formulation (phytantriol (PT)/propylene glycol (PG)/water, 63/27/10, *w*/*w*/*w*) loaded with 20 mg/g MH was proved to be injectable. The precursor formulation can form a cubic phase gel in excess water in 6.97 ± 0.10 s. The results of in vitro drug release suggested the MH presented a sustained release for 4 days. Liquid crystal precursor formulation significantly reduced gingival index, probing depth and alveolar bone loss compared to the model group (*p* < 0.01). Besides, the pathological characteristics of model rats were improved. The results suggested that MH-loaded in situ cubic liquid crystal possessed of sustained release ability and periodontal clinical symptoms improvement. The developed in situ cubic liquid crystal may be a potentially carrier in the local delivery of MH for periodontal diseases.

## 1. Introduction

Periodontitis is a plaque-induced inflammatory condition that affects the periodontium; it is caused by the adherence to tooth surfaces of pathogenic bacterial species organized in complex communities that form biofilms [1]. The pathogens that have been confirmed include *Aggregatibacter actinomycetemcomitans*, *Porphyromonas gingivalis*, *Treponema denticola*, *Tannerella forsythia*, *Prevotella intermedia*, *Parvimonas micra*, *Fusobacterium nucleatum*, *Selenomonas sputigena* and *Eubacterium nodatum*, in the onset and progression of periodontitis [2]. Periodontitis lesions usually harbour a constellation of putative pathogens rather than a single pathogenic species [3]. Periodontitis is a risk factor of some systemic health problems such as vascular inflammation [4], diabetes [5], rheumatoid arthritis [6] and hyperlipidemia [7].

The effective treatment of periodontitis is removing the calculus and plaque by scaling and root planing (SRP). However, due to poor access to the base of deep pockets and anatomical complexities of teeth and furcation involvement, SRP alone may not always result in the complete elimination of pathogens, which results in exacerbation of the disease. This has encouraged the use of antibiotics as an adjunct to mechanical therapy [8]. Orally administered antibiotics suffer from drawbacks due to their systemic effect and also result in lack of effective concentration of the drug at the site of action, resulting in poor patient acceptance. This necessitates the development of alternative localized delivery of drugs [9]. There are multiple options of antimicrobials that can be locally delivered into the mucosa, such as metronidazole, chlorhexidine, minocycline, doxycycline and tetracycline. They show action on both gram-negative and gram-positive organisms. These drugs used in periodontal pockets and can inhibit or eliminate the periodontopathogenic microorganisms as well as modulate the inflammatory response of the tissues [10].

**Figure 1:**
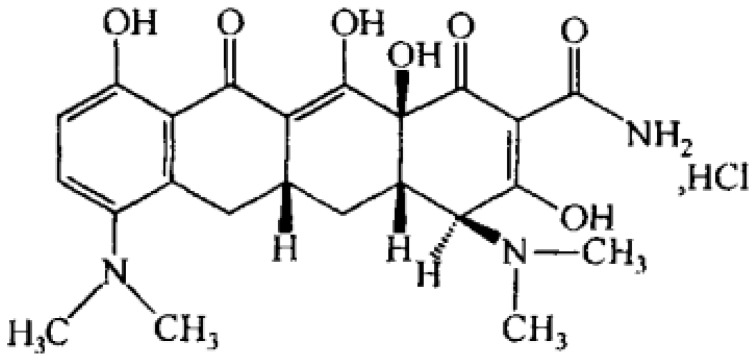
Chemical structure of minocycline hydrochloride.

Minocycline hydrochloride (MH, Figure 1) is a broad spectrum tetracycline antibiotic compared to the other members of the group. It is one of the most active antibiotics against most of the microorganisms associated with periodontal disease. It has the most marked substantivity and shows greater solubility in lipids [11]. The minimum inhibitory concentration (MIC) of MH on Enterobacteriaceae, *Pseudomonas*, *Staphylococcus* and *Candida* isolates from periodontal pockets were 16, 128, 8 and 16 μg/mL [12]. MH shows some advantages in the rehabilitation of periodontitis such as inhibition of collagenase activity, inhibition of bone resorption, promoting the proliferation of periodontal fibroblast and adhesion of periodontal connective tissue [13,14]. Periocline^®^ which is a bio-absorbable sustained local drug delivery system consisting of 20 mg/g MH in a matrix of hydroxyethyl-cellulose, aminoalkylmethacrylate, triacetine and glycerine is commercially available.

The antibiotic release systems used so far in the treatment of periodontitis include in situ gels [15], fibers [16], microparticles [17], nanoparticles [18], films [19] and so on. Recently, in-situ forming implants (ISFI) based on poly(lactic-co-glycolic acid) (PLGA) have been proposed for local periodontitis treatment. These are liquid formulations, which can be easily injected into periodontal pockets, and then (e.g., following solvent exchange) harden to form solid implants with customized geometry. These systems were loaded with antibiotic drugs, namely doxycycline hyclate, metronidazole and minocycline hydrochloride [20]. With Atridox^®^, an ISFI developed by Atrix Laboratories, it has been possible to overcome many drawbacks of the available marketed formulations. The product showed a significant improvement in patient compliance by developing a biodegradable implant that does not require a surgical procedure to place or remove the system [21]. However, there are still some problems in ISFI system, such as solvent safety.

Lyotropic liquid crystals (LLCs) formed by the self-assembly of amphiphilic molecules in a solvent (usually water) have attracted increasingly greater attention in the last few decades. Compared with polymer-based ISFI system, LLC systems has many advantages. The dual polar/apolar structure of LLC systems allows for encapsulation of a wide range of active drugs (i.e., hydrophilic, hydrophobic and amphiphilic) and protect them from hydrolysis and enzymolysis. Phytantriol (PT) contains a saturated aliphatic chain and without ester functional group results in a more stable liquid crystal structure due to the avoidance of the ester hydrolysis reaction [22,23]. Furthermore, PT has been reported to be biodegradable, stable, nontoxic and is easily available in highly pure form [24].

The in situ liquid crystal systems are low viscosity precursor, which are good candidates for injection administration. The precursor could transform into a viscous cubic or hexagonal phase gel in the presence of excess water [25], which facilitate its retention in the periodontal pocket. Researchers have tried to use liquid crystalline as drug carriers in the treatment of periodontal disease. For example, the precursor system of liquid crystalline phase containing propolis microparticles [26] and metronidazole [27] were prepared and characterized. The rheology of systems revealed properties that favored easy injection into the periodontal pocket and subsequent stable retention therein. Furthermore, this systems have been extensively investigated for their ability to sustain the release of bioactives [28]. The purpose of this work was developing a PT-based in situ cubic liquid crystal system containing MH and evaluating the effectiveness on experimental chronic periodontitis when administered as a periodontal pocket topical delivery system.

## 2. Results and Discussion

### 2.1. Development of Precursor Formulations 

**Figure 2:**
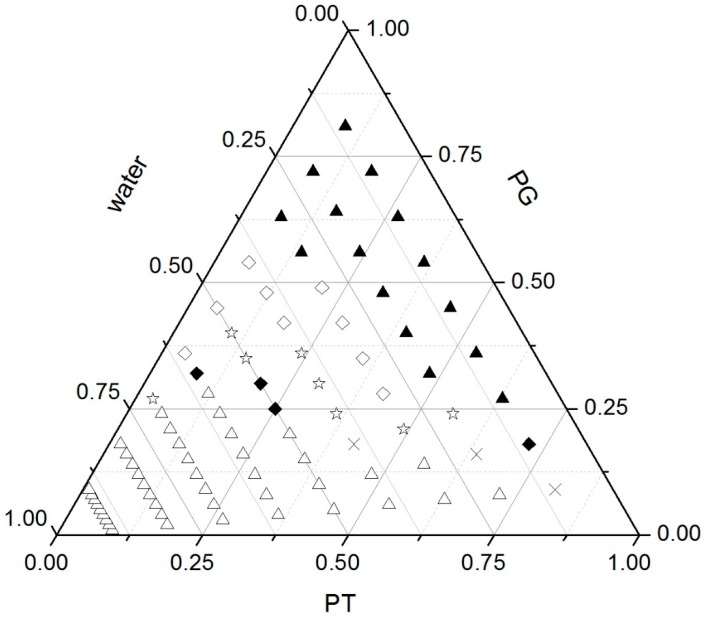
Phase behaviour of the PT-PG-water system. ▲ Isotropic solution; ♢ emulsion; ☆ emulsion + lamellar phase; ♦ lamellar phase; × lamellar + cubic phase; ∆ cubic phase.

A pseudo-ternary phase diagram was constructed by first mixing PT with propylene glycol (PG) in the ratios of 1:9, 2:8, 3:7, 4:6, 5:5, 6:4, 7:3, 8:2 and 9:1 (*w*/*w*). A predetermined amount of each mixture was then transferred into a centrifuge tube and mixed with water at the ratios of 1:9, 2:8, 3:7, 4:6, 5:5, 6:4, 7:3, 8:2 and 9:1 (*w*/*w*) to a total weight of 0.2 g. Each phase was characterized by visual analysis and polarizing light microscopy (PLM). As shown in Figure 2, the sample appeared as a gel in photomicrographs, represented by a dark field, which is a typical characteristic of cubic phase. Malta crosses were observed in the photomicrographs of samples, suggesting the existence of lamellar phase. All mesophase including lamellar phase, cubic phase and the mixture was observed predominantly when PG was present below 30% (*w*/*w*). Samples appearing as emulsions with phase separation after 48 h were characterized as emulsions. The solution samples that showed a dark field under PLM were characterized as isotropic solutions. The isotropic solution formed at a water content of less than 30% and a PG percentage of more than 30%.

Zhang et al. [29] explored the addition of drugs and organic solvent into unsaturated monoglyceride lipid cubic phase, the results of the study showed that the monoglyceride lipid matrix with alcohol, polyethylene glycol, PG or *N*-methyl-2-pyrrolidone can develop low viscosity liquid crystal precursor. In this study, the solubility of MH in PG was higher than that in other solvents. Thus, we developed fluid precursor formulations composed of PT, PG and water. After injection, the precursor formulation needs to absorb gingival crevicular fluid (GCF) to transform into viscous cubic phase gel. GCF is limited in the intraperiodontal pocket. Hence, we chose a flowing isotropic solution phase and lamellar phase, which are injectable and could achieve phase transformation with little additional water for further investigation. Composition of the selected formulations F1–F4 is shown in Table 1.

### 2.2. Physicochemical Characterization

Syringeability, PH value and the ability to form a cubic liquid crystalline gel in situ are very important for the evaluation of an injected formulation, so the physicochemical properties of the formulations F1–F4 are characterized in Table 2. The acceptable pH range for parenteral preparations is 4–9 [30]. The influence of different compositions on phase transformation was also evaluated by minimum volume of water for gelation (V_min_) and gelation time (T_g_). V_min_ and T_g_ decreased with increased water content (formulations F3 and F4) and increased ratio of PT/PG (formulations F1, F2 and F3). Formulation F1 presented minimum values of V_min_ and T_g_ compared to other formulations. In terms of physicochemical properties, formulation F1 is the optimal. However, their release behaviour needs further investigation.

### 2.3. In Vitro Drug Release Studies

**Figure 3:**
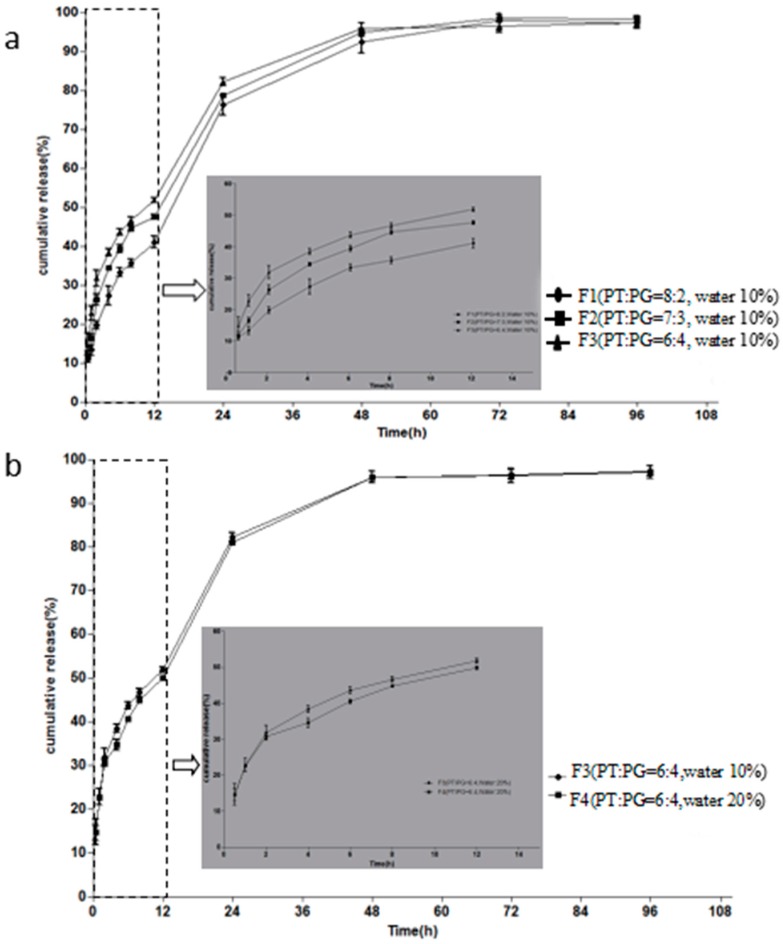
(**a**) Effect of PT/PG ratio on the MH (10 mg/g) release behaviour, (**b**) Effect of water content on the MH (10 mg/g) release behaviour. Inserted panel shows the in vitro release profiles in the first 12 h. Data are presented as mean ± SD (*n* = 4).

In vitro release studies were conducted to investigate the influence of the compositions of formulations on release behaviour. The formulations in Table 1 were chosen for drug release studies, the release profiles are illustrated in Figure 3. Figure 3a presents the plots of the MH percentage released as a function of time from F1, F2 and F3 containing different ratios of PT/PG (8:2, 7:3, and 6:4, *w*/*w*). The release profile of F1 was significantly slower than that formulation F3. No significant difference was observed between F1 and F2. Around 50.0% of drug released in F1–F3 within the first 12 h. Drug sustained release up to 72 h. After 96 h, the release of the drug basically complete, the cumulative release rate was more than 95%. The MH released from F3 and F4 with different water content is illustrated in Figure 3b. When the water content of the formulation decreased from 20 to 10%, the release of MH was slightly lower. Simultaneously, drug was released completely after 96 h. There is some evidence which suggests that the delayed release is due to lower water content and higher PT/PG ratios. Thus, we suggested a hypothesis that MH may be distributed in the water domain of liquid crystal structure.

**Figure 4:**
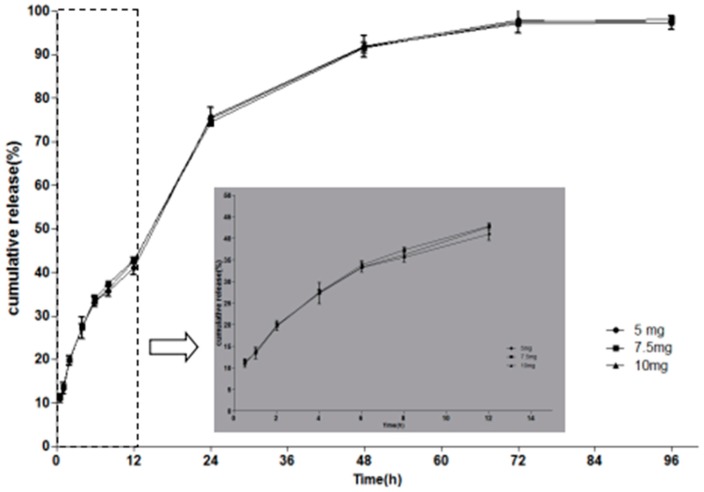
Effect of drug loading on the MH release behaviour. Inserted panel shows the in vitro release profiles in the first 12 h. Data are presented as mean ± SD (*n* = 3).

We further investigated whether the drug loading has an effect on the release behaviour. In this study, a drug precipitation phenomenon was observed in F1 with elevated drug loading. In terms of drug loading, F1 is not suitable as the optimal formulation. Instead, F2 formulations with similar physicochemical properties loaded with 10 mg/g, 15 mg/g and 20 mg/g of MH, were chosen for in vitro release studies. As shown in Figure 4, the release profile of formulation loaded with 10 mg/g of MH was highly similar to the formulation loaded with 15 mg/g and 20 mg/g of MH. There was no significant difference for release rate and cumulative release amount among release profiles. These results indicate that the proportion of drug released was not influenced by the amount of drug loaded into the system. In conclusion, 20 mg/g MH-loaded F2 was the most suitable formulation.

No exact conclusion concerning the effect of drug loading on drug release in vitro is available. The results of this study was consistent with the results of Marilisa et al. who investigated the release behaviour of salicylic cubic phase [31], as well as the results of Jessica et al. who investigated naltrexone-loaded in situ hexagonal liquid crystal [32]. Chen et al. [33] considered that the release of sinomenine hydrochloride-loaded in situ cubic phase increased with the increased of drug loading. However, Qin et al. [34] suggested the release of hydroxycamptothecin-loaded in situ cubic phase decreased with the increased of drug loading. Therefore, we are able to infer that the relationship between in vitro release behaviour and drug loading cannot be generalized. It has been hypothesized that release behaviour may be related to the polarity and solubility of different drugs.

**Figure 5:**
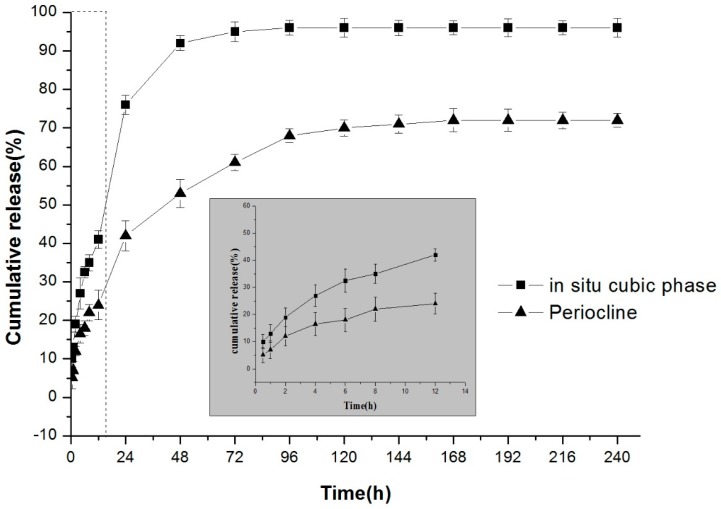
The comparison of the release behaviour of 20 mg/g MH-loaded in situ cubic liquid crystal and Periocline^®^. Inserted panel shows the in vitro release profiles in the first 12 h. Data are presented as mean ± SD (*n* = 3).

A comparative study of in situ cubic liquid crystal and Periocline^®^ was carried out. Figure 5 showed that in situ cubic phase can be sustained release drug for 4 days, and the daily drug release was higher than the MIC of MH. The release rate of cubic phase was significantly faster than that of Periocline^®^. The cumulative release amount of Periocline^®^ was about 70%, while cubic liquid crystal can maintain more than 90% of drug release. The release rate of Periocline^®^ is dependent on the degradation of the matrix material, while the release of the liquid crystal mainly through its unique internal structural features. There are two water channels in the cubic phase structure, and drugs are released from water channel into the environment [35]. Different internal structures lead to different drug release behaviours.

### 2.4. Evaluation of Phase Behavior

**Figure 6:**
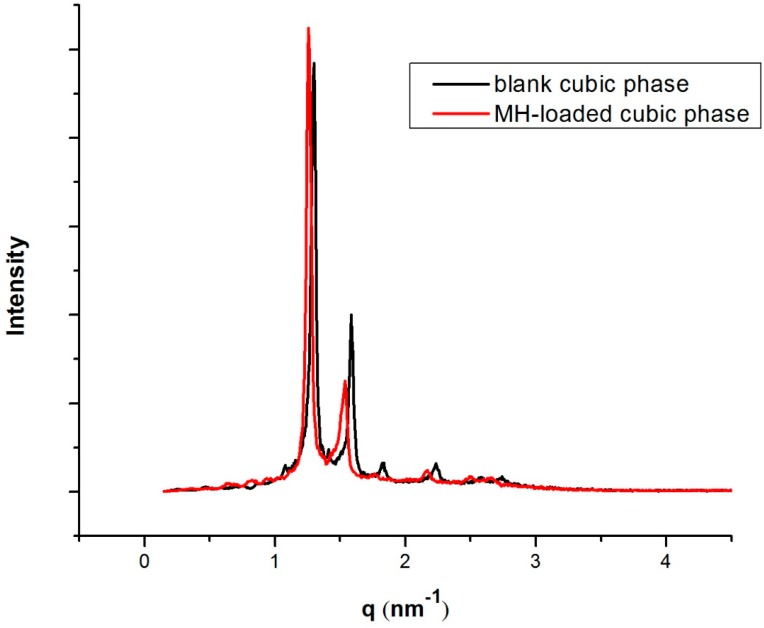
SAXS profiles of blank cubic phase and 20 mg/g MH-loaded cubic phase.

The phase behaviour of optimal formulation was characterized by PLM, small-angle X-ray scattering (SAXS) and rheology methods. Figure 6 shows the SAXS spectra of scattered intensities versus scattering vector q. The same structure was observed in both mesophases, as revealed by the SAXS diffraction peaks in the ratio $\sqrt{2}$:$\sqrt{3}$:$\sqrt{4}$:$\sqrt{6}$. The results demonstrated that blank cubic phase and MH-loaded cubic phase are reversed double diamond bicontinuous cubic phase Pn3m symmetry. As a consequence, the addition of MH did not alter the phase behaviour. 

**Figure 7:**
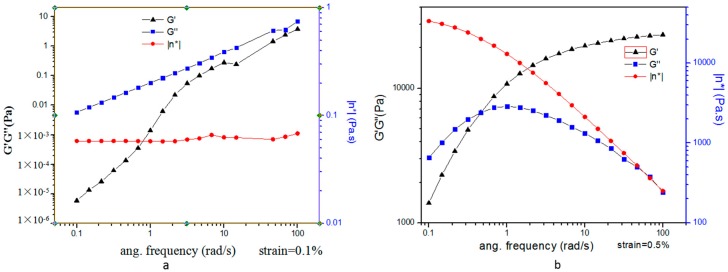
Storage, G′ and loss, G″, moduli as a function of shear frequency. (**a**) MH-loaded precursor formulation. (**b**) MH-loaded precursor formulation in excess water.

Strain-sweep measurements of the precursor formulation shown a linear relation between the shear stress and the shear rate, which is a characteristic Newtonian behaviour. The liquid crystal precursors have a flow property that renders them easy to apply to the required site. The oscillatory frequency sweep was carried out on the precursor formulation and in situ liquid crystal in excess water. The storage modulus G′ and the loss modulus G″ were plotted against frequency, and representative rheograms are presented in Figure 7.

The precursor formulation were found to be more viscous than elastic (G″ > G′), indicating “liquid-like” behaviour. In situ liquid crystal in excess water were found to be more viscous than elastic (G″ > G′) at a low frequency and more elastic than viscous (G′ > G″) at a high frequency. The results showed that cubic phase was a viscoelastic system and presented “gel-like” behaviour. Hence, the “liquid-like” behaviour of precursor formulation is more benefit to inject for intra-periodontal pocket administration. The “gel-like” behaviour of phase transition formulation keep drug in the periodontal pocket and sustained release.

### 2.5. In Vivo Pharmacodynamics Studies

**Figure 8:**
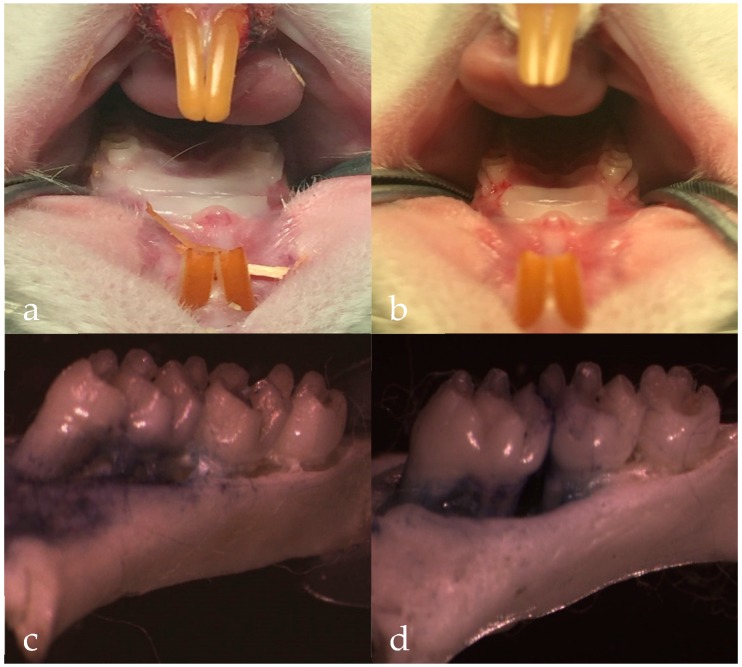
The photographs of gingival tissue: (**a**) normal group, (**b**) model group. The photographs of alveolar bone: (**c**) normal group, (**d**) model group, magnification ×12.5.

**Figure 9:**
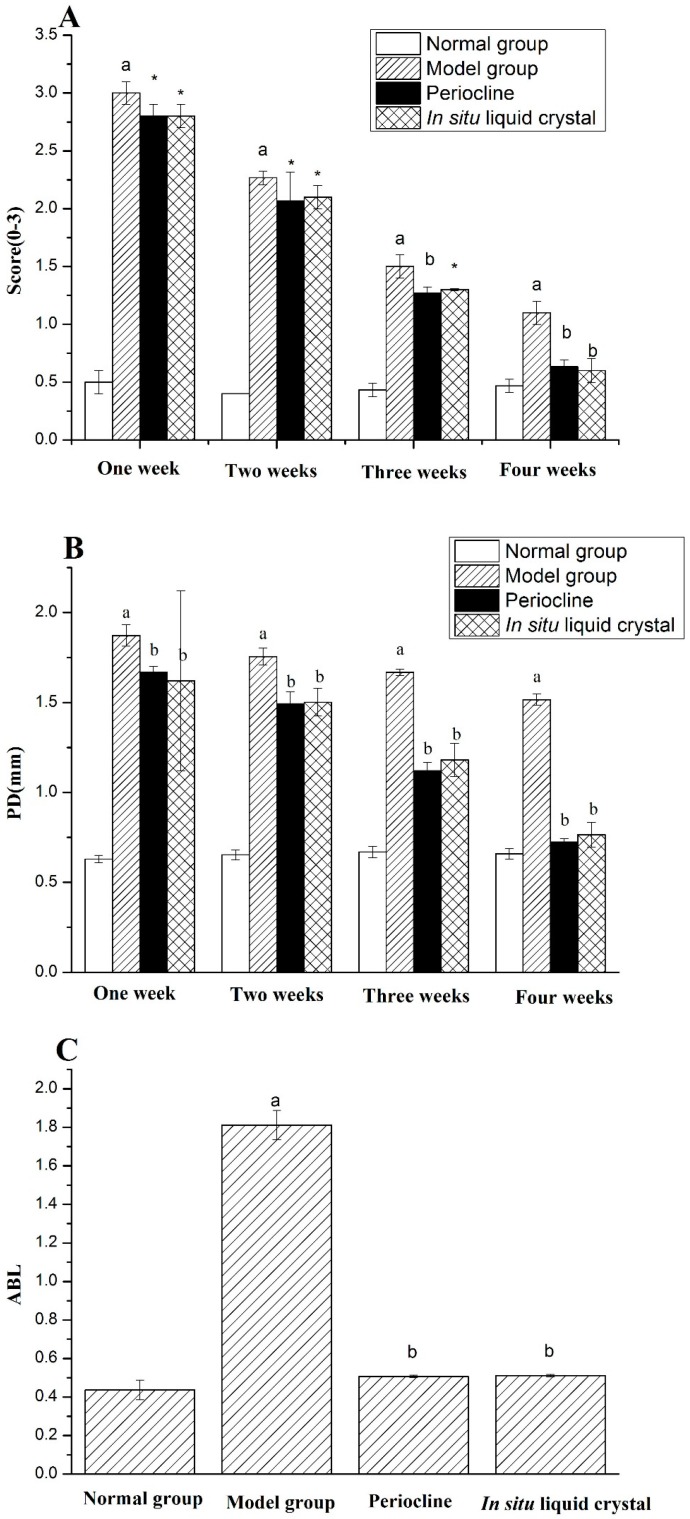
The related indicators of four groups after 4 weeks treatment: (**A**) Scores of gingival index (GI), (**B**) Pocket depth (PD) and (**C**) Alveolar bone loss (ABL). Values indicate the mean ± SD (*n* = 5). ^a^ *p* < 0.01, compared with normal group; * *p* < 0.05, compared with model group, ^b^ *p* < 0.01, compared with model group.

High glucose feeding [36], silk ligation of animal teeth [37] and periodontal local inoculation of suspected pathogens [38] can be all used to induce periodontitis. The latter two methods obtain the local pathological manifestations of periodontitis by locally adding periodontal stimulants. High glucose can weaken the ability of cell migration, weaken the healing ability of periodontal tissues and affect the inflammatory secretion of gingival epithelial cells. Meanwhile, the expression of Toll-like receptor 4 (TLR4) and Interleukin 6 (IL-6) in human gingival epithelial cells can be upregulated Periodontitis is a multifactor-induced disease, and the current model of periodontitis in rats often combines two or more methods in model rats, In this study, the use of silk ligation combined with high-sugar feeding method was used and after 10 weeks, the typical symptoms of periodontitis were observed, including gingival edema, gingival soft and depressed alveolar bone resorption (Figure 8). This suggested that periodontitis model was constructed successfully. After 4 weeks treatment of Periocline^®^ and in situ cubic liquid crystal, the scores of gingival index (GI), probing depth (PD) and alveolar bone loss (ABL) of each group is graphically illustrated in Figure 9. The model group showed significant higher GI, PD, and ABL levels compared to the normal group (*p* < 0.01). There was significant reduction of inflammatory symptoms in Periocline^®^ group and in situ cubic liquid crystal group compared with model group (*p* < 0.01). Likewise, the values of GI, PD, and ABL are all close to the normal group in the first four weeks.

**Figure 10:**
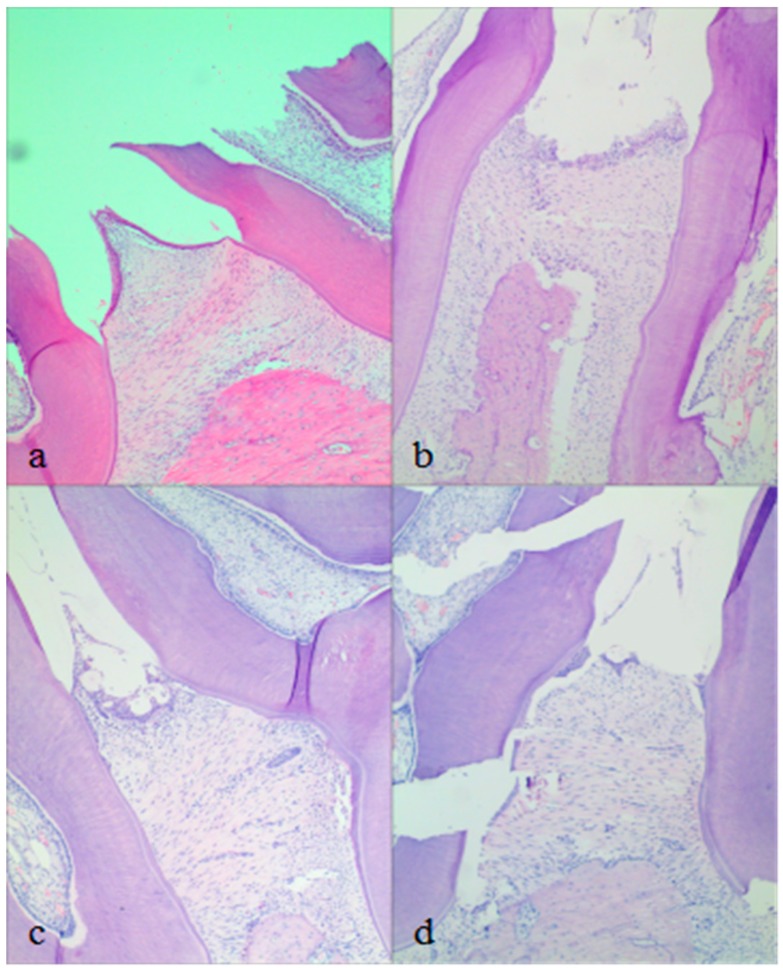
Histopathological observation: (**a**) normal group, (**b**) model group, (**c**) Periocline^®^ group (after 4 weeks treatment), (**d**) MH-loaded in situ cubic liquid crystal group (after 4 weeks of treatment). H&E × 100.

Histopathological results of the periodontal tissue are depicted in Figure 10. The normal group showed conical gingival papilla, neatly arranged gingival collagen fibers and complete gingival epithelium. The junctional epithelium is attached to cemento–enamel junction (CEJ), and the alveolar bone crest with smooth morphology has no resorption. The periodontal tissue showed signs of chronic inflammation in the model group. Epithelial erosion, gingival papilla depression, bone resorption of depressed type, destruction of alveolar bone, collagen fiber derangement and resorption of cementum were found. The junctional epithelial detachment from the CEJ to the root proliferative shift. More osteoclasts appear in the alveolar bone crest. After 4 weeks treatment, the two medicated groups showed different degrees of repair. For the Periocline^®^ group, the gingival epithelium was repaired slightly and re-attached to CEJ. Neatly arranged gingival collagen fibers and smooth alveolar bone crest were basically restored. Depressed alveolar bone resorption was disappeared nearly. Compared with Periocline^®^ group, in situ cubic liquid crystal presented the similar effect. Periocline^®^ as the positive control, it can be confirmed that MH in situ cubic liquid crystal has an therapeutic effect on the restoration of the gingival epithelium, gingival collagen fibers and the alveolar bone. 

The liquid crystal system presented in this work exhibits its own unique advantages while exerting similar effects on periodontitis compared to other systems. The liquid crystal system can obtain properties in relation to the periodontal administration without using various additives and toxic solvents, such as sensitive solution–gel phase transition, unique nanostructures, gel strength, good adhesiveness and suitable mechanical properties. These properties can overcome a particular problem of poor retention at the site of application of many drug delivery systems for periodontal pockets.

## 3. Materials and Methods 

### 3.1. Materials

Phytantriol (3,7,11,15-tetramethyl-1,2,3-hexadecanetriol, PT, GC > 95%) was obtained from Tokyo Chemical Industry Co., Ltd. (Shanghai, China). Minocycline hydrochloride was provided by Wuhan Sheng Tianyu Biotechnology Co., Ltd. (Wuhan, China). Periocline^®^ was obtained from New-Era Co., Ltd. (Osaka, Japan). PG was purchased from Nanjing Chemical Reagent Co., Ltd. (Nanjing, China). Dialysis bags (molecular weight cut off: 14,000 Da) were obtained from Yuanye BioTechnology Co., Ltd. (Shanghai, China). Purified water used in all experiments was processed using a Milli-Q system (Millipore, Bedford, MA, USA). All other reagents were of analytical or pharmaceutical grade. 

### 3.2. Preparation of Precursor Formulations

The precursor formulations were prepared by mixing PT, PG and water. PT was gently melted at 60 ± 0.5 °C followed by the addition of the required amount of PG at the same temperature. The MH was dissolved in the PG. The mixture was vortex-mixed homogeneously, and the appropriate quantity of prewarmed water at the same temperature was added into the mixture. Then, the mixture was homogeneously vortex-mixed. The formulations were finally sterilized by filtration through a 0.22 μm filter and sealed in ampoules to equilibrate for 72 h before any experiments.

### 3.3. Evaluation of Phase Behaviour

#### 3.3.1. PLM

The precursor formulations and the gel obtained in excess water were macroscopically characterized by visual observation and examined microscopically under a polarized light microscope (XP-330C, Cai Kang Optical Instrument Co., Ltd., Shanghai, China) at room temperature.

#### 3.3.2. SAXS Measurements

Unloaded and MH-loaded precursor fluid formulations and the gel obtained in excess water were evaluated by an SAXSess mc2 SAXS (Anton Paar, Graz, Austria) equipped with a sealed X-ray tube (Cu-anode target type) producing Ni-filtered Cu Ka radiation with a wavelength of *λ* = 0.15418 nm. The voltage was set to U = 40 kV with an anode current of I = 50 mA. The optics and sample chamber were under vacuum to minimize air scatter. Measurements were performed at 37 °C with measurement time setting t = 15 min. And samples were equilibrated for 10 min prior to measurements. 

#### 3.3.3. Rheological Measurements

Rheological measurements were carried out with a stress controlled rheometer AR-2000ex (TA Instruments, New Castle, DE, USA) in the flow and oscillatory modes. A cone-plate sensor with a diameter of 20 mm and a cone angle of 1° was used. Measurements were performed after a period of 2 min to allow for the stress relaxation. The linear viscoelastic domain of a material was determined via an oscillatory stress sweep at a fixed frequency (1 Hz) before carrying out the oscillatory measurements. Strain-sweep measurements were carried out over a range of strain (0.01–100%). A constant strain was chosen in the linear viscoelastic domain, the samples were subjected to frequency sweep measurements at 25 ± 0.1 °C for the MH-loaded precursor formulations and 37 ± 0.1 °C for the gel obtained in excess water over a frequency range of 0.01–100 rad·s^−1^. The viscoelasticity of the sample before and after phase transition was characterized in terms of the elastic modulus G’ and the loss modulus G″. Flow-sweep measurements were performed on MH-loaded precursor formulations over a range of shear rates (1–200 s^−1^) at temperature of 25 ± 0.1 °C.

### 3.4. Physicochemical Characterization

#### 3.4.1. Evaluation of Syringeability and Determination of pH Value

In this work, syringes equipped with a modified plastic pipette tip were chosen to evaluate the syringeability of the formulations at room temperature. The inner diameter of injection head is about 0.5 mm. The pH values of chosen formulations were determined by a SevenMuti type multi-tester (Mettler Toledo, Shanghai, China).

#### 3.4.2. Determination of the V_min_

The V_min_ of the chosen formulations was determined by the magnetic stirring method [39]. First, 0.1 g of the MH-loaded precursor formulation was aliquoted into a 5 mL centrifuge tube, a magnetic bar (10 × 6 mm) was added into the centrifuge tube. The centrifuge tube was incubated in a water bath at 37.0 ± 0.5 °C for 5 min with the magnetic stirring speed of 30 rpm. Then, 10 μL of water was added into the centrifuge tube every 1 min until the magnetic bar completely stopped moving due to gelation. The total volume of the water added into the centrifuge tube was determined to be the V_min_ of the sample [24].

#### 3.4.3. Determination of the T_g_

The internal and external substances of periodontal pocket was easily removed by the GCF flow, and rapid phase transition should be achieved when in situ cubic liquid crystals in combination with the required amount of GCF, so the T_g_ of the chosen formulations was also determined by the magnetic stirring method. The operation was the same as the determination of V_min_, whereby excess water was added into the centrifuge tube. The time when the magnetic bar completely stopped moving due to gelation was determined to be the T_g_ of the sample.

### 3.5. In Vitro Drug Release

In vitro release of MH was determined in triplicate by using a dialysis membrane diffusion method [40]. Briefly, the formulations (0.5 g) were placed separately in 6 cm dialysis bags. The dialysis bags were then closed and immersed into 6 ml PBS (PH 7.2–7.4) in centrifuge tube, which were further placed in a horizontal shaker (37.0 ± 0.5 °C, 60 rpm). Sodium azide (0.02% *w*/*w*) was added to the dissolution medium to prevent bacterial contamination. After 0.5, 1, 2, 4, 6, 8, 12, 24, 48, 72, 96, 120, 144, 168, 192, 216 and 240 h, all of dissolution medium was withdrawn from each vessel and immediately replaced with 6 ml fresh dissolution medium each time. The amount of MH released was analysed by UV spectrophotometry at 274 nm (UV-L5S, Precision Science Instrument Co., Ltd., Shanghai, China). Release amount and cumulative release rate were calculated by absorbance at predetermined time points.

### 3.6. In Vivo Pharmacodynamics Studies

All the animal studies were approved by the Animal Ethical Committee of Anhui University of Chinese Medicine (Ethic approval No. KC: 027-15, KC: 027-16). The experiments were conducted in accordance with the guidelines of the Laboratory Animal Center of Anhui University of Chinese Medicine. SPF rats (3 months old, 300 ± 20 g) were divided into four groups: normal group, model group, MH-loaded in situ cubic liquid crystal group and Periocline^®^ group, five rats in each group.

A rat model of chronic periodontitis was implemented by a combination of the thread ligation method [41] and high glucose feeding. The animals were anesthetized with 5% chloral hydrate (350 mg/kg) by intraperitoneal injection before surgery. Firstly, the crevice, which is located between in the first molar and the second molar was established by slowly tugging with 4-0 surgical suture. Then, the neck of the tooth was ligated using double circle silk thread and the ligature was placed into the gingival sulcus. Sucrose solution (100 g/L) was administered as drinking water during the modeling period. The thread was checked three times a week, and the whole process was maintained for 10 weeks.

Two groups of rats were tested by intra-pocket administration of either in situ cubic liquid crystal or Periocline^®^ once a week and sustained for 4 weeks. The food and water of the four groups of rats were supplied normally. GI, PD and ABL were measured every week. GI score: 0 = healthy, 1 = slight, 2 = moderate and 3 = severe. PD was monitored using periodontal probe in the tongue side and buccal side of the far, middle and near, respectively. Rats were sacrificed and maxillary third molar alveolar bone tissue was excised to determine ABL. The tissue immersed in 1 mol/L NaOH solution for 24 h. Then soft tissue was removed and Loeffler’s Methylene Blue disseminated to the CEJ. The tissue is placed under the stereo microscope, 12.5 times magnification and measured the distance from the CEJ to the alveolar crest. Mean value of tongue side and buccal side of the far, middle and near were taken as the ABL. Histopathological experiments were also conducted on the periodontal tissue of the upper and lower teeth with three molars. The tissue was fixed in 4% polyformaldehyde solution for 24 h, following by moving into decalcifying fluid for 4 weeks and decalcifying fluid was replaced the next day. Finally, the tissue was observed using light microscopy after hematoxylin and eosin (H&E) staining.

### 3.7. Statistical Analysis

Statistical analysis was carried out by the SPSS statistical software (23.0 version, SPSS Inc., Chicago, IL, USA). Each experiment was performed in triplicate and all data were expressed as mean ± standard deviation (SD). One-way analyses of variance were performed for evaluation of the results. *p*-values below 5% (*p* < 0.05) were considered statistically significant.

## 4. Conclusions

The in situ liquid crystal delivery system presented in this work was found to possess the needed low viscosity, sensitive solution-gel phase transition and favourable physicochemical properties. The formulation showed a typical characteristic of cubic phase in excess water by PLM, SAXS and rheological measurements. The in vitro release experiments showed that the MH-loaded in situ liquid crystal presented higher cumulative releases than Periocline^®^, and the formulation be able to sustain the drug release for 4 days. The pharmacodynamics results indicated that MH-loaded in situ cubic liquid crystal had therapeutic effects on periodontitis. This system provided a successful and effective drug delivery method and may be used as a potential carrier in the local delivery of MH for periodontal diseases.

## Figures and Tables

**Table 1:** The compositions of each MH formulations.

Formulation	PT (%)	PG (%)	Water (%)	MH (mg/g)
F1	72	18	10	10
F2	63	27	10	10
F3	54	36	10	10
F4	48	32	20	10

PT: phytantriol; PG: propylene glycol; MH: minocycline hydrochloride.

**Table 2:** Physicochemical properties of F1–F4 formulations.

Formulations	Syringeability	pH	V_min_ (µL)	T_g_ (s)
F1	Injectable	5.22	38.12 ± 0.17	4.40 ± 0.09
F2	Injectable	5.17	60.09 ± 0.09	6.97 ± 0.10
F3	Injectable	5.11	74.83 ± 0.22	11.70 ± 0.14
F4	Injectable	5.25	65.5 3 ± 0.12	7.09 ± 0.20

V_min_: minimum volume of water for gelation; T_g_: gelation time. Data expressed as mean ± SD, *n* = 3.
